# Elbow Joint Stiffness Functional Scales Based on Hill’s Muscle Model and Genetic Optimization

**DOI:** 10.3390/s23031709

**Published:** 2023-02-03

**Authors:** Marija Radmilović, Djordje Urukalo, Milica M. Janković, Suzana Dedijer Dujović, Tijana J. Dimkić Tomić, Maja Trumić, Kosta Jovanović

**Affiliations:** 1 Institute Mihailo Pupin, University of Belgrade, Volgina 15, 11060 Belgrade, Serbia; 2 School of Electrical Engineering, University of Belgrade, Bulevar Kralja Aleksandra 73, 11120 Belgrade, Serbia; 3 Clinic for Rehabilitation “Dr. Miroslav Zotović”, Faculty of Medicine, University of Belgrade, 11000 Belgrade, Serbia

**Keywords:** agonist–antagonist, electromyography, genetic algorithm, passive joint structure, Wolf Motor Function Test

## Abstract

The ultimate goal of rehabilitation engineering is to provide objective assessment tools for the level of injury and/or the degree of neurorehabilitation recovery based on a combination of different sensing technologies that enable the monitoring of relevant measurable variables, as well as the assessment of non-measurable variables (such as muscle effort/force and joint mechanical stiffness). This paper aims to present a feasibility study for a general assessment methodology for subject-specific non-measurable elbow model parameter prediction and elbow joint stiffness estimation. Ten participants without sensorimotor disorders performed a modified “Reach and retrieve” task of the Wolf Motor Function Test while electromyography (EMG) data of an antagonistic muscle pair (the triceps brachii long head and biceps brachii long head muscle) and elbow angle were simultaneously acquired. A complete list of the Hill’s muscle model and passive joint structure model parameters was generated using a genetic algorithm (GA) on the acquired training dataset with a maximum deviation of 6.1% of the full elbow angle range values during the modified task 8 of the Wolf Motor Function Test, and it was also verified using two experimental test scenarios (a task tempo variation scenario and a load variation scenario with a maximum deviation of 8.1%). The recursive least square (RLS) algorithm was used to estimate elbow joint stiffness (*Stiffness*) based on the estimated joint torque and the estimated elbow angle. Finally, novel *Stiffness* scales (general patterns) for upper limb functional assessment in the two performed test scenarios were proposed. The stiffness scales showed an exponentially increasing trend with increasing movement tempo, as well as with increasing weights. The obtained general *Stiffness* patterns from the group of participants without sensorimotor disorders could significantly contribute to the further monitoring of motor recovery in patients with sensorimotor disorders.

## 1. Introduction

Functional assessment is a basic and indispensable component of neurorehabilitation that identifies sensorimotor deficits in patients, determines priorities, and plans therapeutic protocols, i.e., it monitors the effects of therapy. Clinical scales such as the Fugl-Meyer Assessment (FMA) and the Wolf Motor Function Test (WMFT) are frequently used to assess motor functionality in patients after stroke [1,2]. However, clinical scales are not sensitive enough to capture the quality of sensory and motor performance. To improve our understanding of the mechanisms that drive motor recovery, we need to distinguish between ‘true neurological repair’ (i.e., restitution), in which neurological impairments are restored towards normal, and behavioral compensation strategies [3]. Technological progress and development have enabled the production of various portable and built-in sensors that accurately record kinetic and kinematic parameters, as well as additional parameters capable of objective assessment of the sensorimotor function of the upper extremities [4]. New devices and technologies that contain different sensor systems for measuring trajectories, joint angles, forces, and electromyography (EMG) signals, as well as systems that provide estimations of non-measurable variables (such as muscle effort/force and joint mechanical stiffness or impedance) are of great use for precise kinetic and kinematic analysis of motor behavior. Kinematic data can be obtained during performance of a specific functional task or with specially designed non-functional assays, and depending on the analysis, it is possible to determine whether a given movement is compensatory or becoming more similar to a normal movement. Recovery trials need to consider serially applied kinematic/kinetic measurements alongside clinical assessments to distinguish between restitution and compensation [5]. 

Among non-measurable variables, the human ability to adequately generate an interaction force is recognized as one of the basic parameters for the analysis of human movement dynamics and its application in neurorehabilitation. Pathological conditions, such as post-stroke, lead to a reduction in joint stiffness and force due to the improper activation of antagonist muscle pairs. While several studies have investigated how to detect the rotational stiffness of lower-limb joints during locomotion, exploiting the cyclicity of gait or implementing musculoskeletal models driven by muscle activity, less attention has been given to the real-time estimation of upper limb stiffness modulation during non-cyclic movements [6].

Basic approaches have been proposed to estimate joint stiffness and force during human arm movement [7]. Stiffness estimation for short-range human arm movement with small variations in the interaction force can be performed using linear models. On the other hand, stiffness estimation for long-distance hand movements with large variations in the forces of interaction with the environment involves nonlinear models using musculoskeletal modeling [8,9].

A common method for estimating joint stiffness during small movements of the human arm using a linear model was proposed by Perreault [10,11]. This approach is based on applying stochastic perturbation to the human hand using a robotic manipulator and measuring the restoring forces imposed by the hand movement. Ajoudani et al. extended the research of Perreault et al. [10,11] to include muscle activation using EMG signals as additional parameters to assess endpoint stiffness. They concluded that endpoint stiffness of the human arm is related to the muscle co-contraction index and arm configuration. In [12], the Jacobian matrix of the human arm and the co-contraction index of the dominant antagonist muscle pair define the endpoint stiffness. Changing the activation of the dominant muscles directly affects the volume of the stiffness ellipsoid, while changing the position of the arm affects the direction of its axis. The identified endpoint stiffness model of the human arm in real time is used in the tele-impedance of the robot. In a continuation of this research, Ajoudani et al. [13] presented the application of new models of the musculoskeletal system of the human arm based on the muscular Jacobian and the synergistic contribution of muscle activities (the long heads of the biceps brachii and triceps brachii) to estimate the endpoint stiffness. The disadvantage of this approach is that it does not include the joint moment within the muscular Jacobian matrix. The evaluation of the complete joint stiffness matrix is incomplete because the endpoint stiffness from the end effector space needs to be transferred to the larger joint space. To identify the actual total joint stiffness of the human hand, they developed a new technique based on nullspace stiffness [14]. It is a complex two-stage identification procedure where, in the first stage, shoulder–hand rotational perturbations are applied with wrist fixation, and in the second stage, translational and rotational perturbations are applied without a wrist brace. On the other hand, Stanev et al. [15] analyzed the problem of redundancy at the muscle level. They proposed a method to estimate the endpoint and joint stiffness of the redundant musculoskeletal model for any arm movement. Since the CNS uses different strategies to coordinate muscles during arm movement, they proposed a nullspace solution for muscle redundancy and calculation of the joint stiffness according to defined tasks and muscle constraints. The presented results are based on simulation of the arm and leg. In the first part of the analysis, a simplified arm model with three degrees of freedom and nine muscles without joint nonlinearity was used, and in the second part of the analysis, the existing OpenSim model was used without any improvements. In [16], a new model was constructed based on the main axes of the stiffness ellipsoid and their lengths, i.e., the Eigen decomposition of the stiffness matrix, based on the arm configuration. Eigen decomposition was much easier compared with Jacobians, especially the muscle Jacobian. They defined four parameters that are closely related to muscle strength and skeletal dimensions. In this way, the stiffness calculation was simplified and allowed for personalized matching of an individual’s physical interaction characteristics. An average model error of about 20% existed due to the neglect of the influence of external forces and their assumption on muscle synergy. This approach is not suitable for other applications that are more sensitive to variation in the stiffness value. Most studies are based on a perturbation experiment, where joint stiffness is estimated from a linear model using small displacement and small variations in muscle activity. This approach is not suitable for estimating joint stiffness in real-world scenarios. Since the human arm behaves nonlinearly on the large scale of hand movement and interaction force represented in real life, nonlinear modeling of the human hand is required [17].

Nonlinear models of the human arm require modeling of the musculoskeletal system, which is predominantly based on Hill’s muscle model and the passive joint structure model [18,19]. This model uses the muscle activation of the antagonistic muscle pair as the input parameter, whereby the muscle activation is estimated using EMG signals of antagonistic muscles during targeted movement. The values of the Hill’s muscle model and passive joint structure model parameters of upper extremities have been shown through several studies [20,21,22,23,24,25,26,27,28], but none of these publications provide a full list of range values of the parameters.

The research performed in this paper takes into account the nonlinearity of human muscles and considers a stiffness estimation method based on Hill’s muscle model and the passive joint structure model, resulting in a complete list of verified model parameters during the elbow flexion/extension task, as well as elbow stiffness curves (so-called functional scales) for different task tempos and loads. It is worth noting that our approach provides continuous estimation of stiffness in time, which is highly beneficial for human–robot interaction tasks [8,29] and for planning the stiffness trajectory of an artificial variable-stiffness elbow joint that is used as a prosthesis [30]. This paper provides a feasibility study for a general assessment methodology that overcomes one-size-fits-all challenges through the application of a genetic algorithm (GA) for subject-specific elbow model parameter prediction. We used an approach based on a commonly accepted joint/muscle physical model [8,18,19,20,21,22,23,24,25,26,27,28], where we derived a subject-specific GA-based model, and then, estimated joint stiffness based on experiments using wearable sensors. This approach gives us the ability to predict and measure stiffness and other non-measurable quantities based on the model, even when measurements are not directly available. The GA-based methodology was applied to a population without sensorimotor disorders during a “Reach and retrieve” task of the Wolf Motor Function Test—WMFT (modified to include elbow extension in addition to elbow flexion) to examine the feasibility of the proposed objective functional assessment, considering that the same methodology could be applied to patients with sensorimotor disorders and upper extremity weakness in the future. The main contributions of the paper are as follows: (1) identification of the range of the Hill’s muscle model and passive joint structure model parameters using an elbow flexion/extension task in a group of ten healthy volunteers without sensorimotor disorders based on GA, providing of a full list of the range values of the parameters; (2) verification of the identified model parameters; (3) continuous-time elbow joint stiffness estimation based on verified subject-specific model parameters; (4) the definition of novel scales for assessment based on elbow joint stiffness estimation in scenarios of different task tempos and elbow loads.

## 2. Materials and Methods

### 2.1. Participants

Ten healthy volunteers without sensorimotor disorders participated in the study: 5 males (age 33.6 ± 4.27) and 5 females (age 28 ± 9.35). The participants verbally confirmed that they had no previous injuries to the elbow joint, nor did they currently feel any discomfort. Before the experiment, the selected body segment parameters (BSP) of each participant were measured using the Hanavan model of the human body [31] (Table 1).

Based on BSP parameters, we estimated, for each participant, the mass (m) of the forearm–hand segment, the distance from the elbow joint axis to the center of the forearm mass (r_cm_), and the moment of inertia (M) of the forearm-hand segment. The estimated parameters were used as input parameters for Hill’s muscle model and the passive joint structure model (see Section 2.4.1 and Section 2.4.2).

### 2.2. Experiment Description

**Figure 1:**
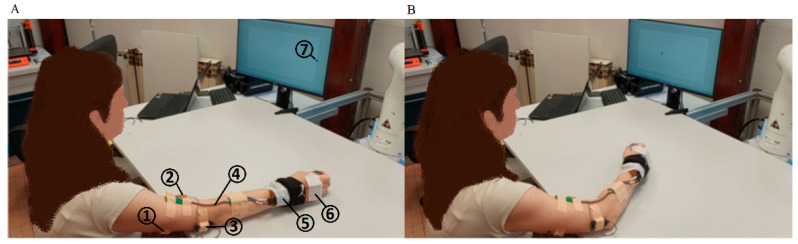
Experimental setup: (**A**) initial position of the arm; (**B**) the position of the arm during the motion. 1—EMG electrodes located on triceps brachii long head muscle, 2—EMG electrodes located on biceps brachii long head muscle, 3 and 5—IMU units, 4—goniometer, 6—wrist fixation, 7—feedback display of the movement tempo.

At the beginning of the experiment, the participant was sitting on a chair at a table with their arm extended in front of them. The arm was supported by the elbow on the table so that only the forearm was allowed to move in the horizontal plane. The wrist joint was immobilized to avoid its independent movement. This position was taken as the initial position of the arm (zero angles in the elbow, see Figure 1). The monitor for the feedback display of the movement tempo was positioned in front of the participant.

The experimental protocol of the performed study was based on the “Reach and retrieve” task of WMFT (task no. 8, where the participant attempts to pull the weight across the table by flexing the elbow) [32]. In the performed study, the “Reach and retrieve” task was modified, with additional extension of the elbow with the same load as in the flexion.

All participants were subjected to the same experimental conditions. Each participant was instructed to move their hand while holding the weight from the starting position to a position that corresponded to the maximum flexion of the user’s elbow (the range of elbow motion was approximately from 0° to 150°), i.e., touching the chest with the hand. The far-right position of the “moving dot” on the feedback monitor corresponded to an outstretched human arm (the initial position), while the far-left position corresponded to maximum flexion at the elbow. The participants were instructed to avoid pushing the weight against the table and lifting the weight. Friction between the weights and the table was ignored due to the rigid, smooth finish and different materials. The weight was made of stainless steel and the table had a veneer top.

The overall performed task, the modified task 8 of WMFT (WMFT8_m_), included: (1) attempting to push the weight across the table by flexing the elbow and (2) attempting to push the weight across the table by extending the elbow.

During the task activity, the Trigno^TM^ Avanti (Delsys, Natick, MA, USA) system was used for the data acquisition of: (1) surface EMG activity of two antagonistic muscle pairs of the upper arm (triceps brachii long head and biceps brachii long head muscle) and (2) the angle in the elbow. Trigno Avanti Duo EMG units (Delsys, Natick, MA, USA) were used for EMG recordings and a goniometer (Biometrics, Ladysmith, VA, USA) with Trigno Avanti Goniometer Adapter Sensor (Delsys, Natick, MA, USA) was used for angle measurement. The positions of all sensors are shown in Figure 1. EMGworks Acquisition software (Delsys, Natick, MA, USA) was used for simultaneous data acquisition from all sensors. The sampling rate for data acquisition was approximately 2000 Hz.

The overall experimental scenario included the following phases:

- Maximum voluntary contraction (MVC) data acquisition for the triceps brachii long head and biceps brachii long head muscle against static resistance [33,34,35,36]. The recorded values of the MVC were used in all experiments for normalization of the recorded EMG signals (see Section 2.3).
- Training phase. In the beginning, the participant’s arm was in the initial position (Figure 1A). The participant repeated the WMFT8_m_ rotation (while pulling the 0.5 kg weight) of the elbow joint following the tempo of the “moving dot” shown on the feedback display. The tempo of the “moving dot” was set to 15 beats per minute (bpm), 30 bpm, 45 bpm, and 60 bpm and it was changed for each 10 WMFT8_m_ movement, respectively. This phase was used for estimating the parameters of Hill’s model (see Section 2.4.1) by GA, as described in Section 2.4.3.
- Task frequency variation experiment (E1). In the beginning, the participant’s arm was in the initial position (Figure 1A). The participant repeated the WMFT8_m_ rotation (while pulling the 0.5 kg weight) of the elbow joint following the tempo of the “moving dot” shown on the feedback display. The tempo of the “moving dot” was set to 15 bpm, 30 bmp, 45 bmp, and 60 bmp. The participant repeated the WMFT8_m_ movement for each tempo 10 times in three series. The resting pause between series was 1 min. The resting pause between different tempos was 5 min.
- Load variation experiment (E2). In the beginning, the participant’s arm was in the initial position (Figure 1A). The participant repeated the WMFT8_m_ rotation of the elbow joint following the tempo of the “moving dot” shown on the feedback display. The tempo of the “moving dot” was set to 30 bmp. The participant repeated the same WMFT8_m_ movement with different “pulling” weights: 0.25, 0.5, 0.75, and 1 kg. The participant repeated the WMFT8_m_ movement for each weight 10 times in three series. The resting pause between series was 1 min. The resting pause between different weights was 5 min.

The experimental design was approved by the ethical review board of the University of Belgrade—School of Electrical Engineering. Participants were well-informed about the noninvasive protocol, and they signed informed consent forms.

### 2.3. EMG Processing and Muscle Activation Dynamics

**Figure 2:**
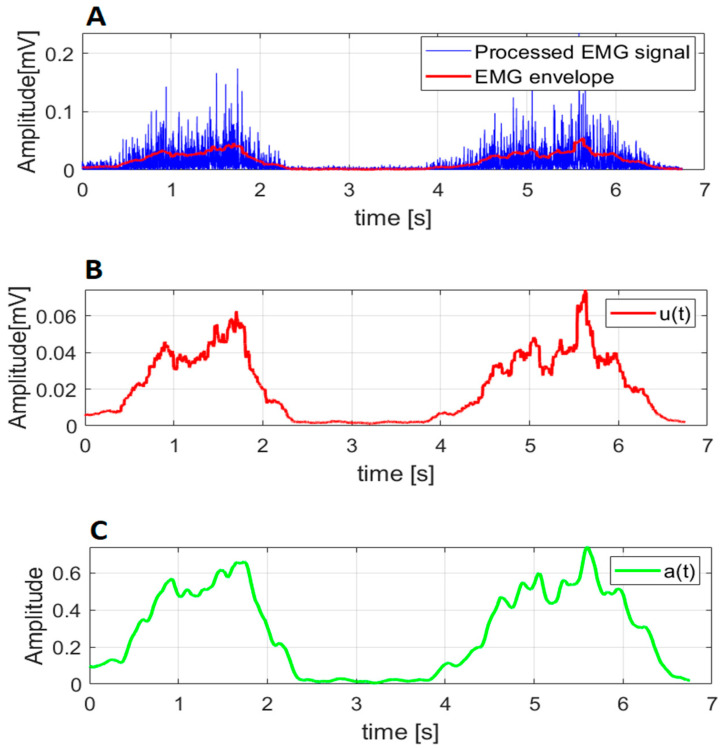
Muscle activation estimation: (**A**) Processed EMG signal (blue line) and EMG envelope (red line); (**B**) neural activation u(t); (**C**) muscle activation a(t) obtained using Equation (1).

The acquired EMG and MVC signals from antagonistic pairs were processed to achieve muscle activation. First, the EMG and MVC signals were filtered using the Butterworth bandpass filter (10–500 Hz, order = 5) and the Butterworth Notch filter (48–52 Hz, order = 5). The filtered EMG and MVC signals were rectified, giving the Processed EMG signal (Figure 2, left) and Processed MVC signal. The EMG and MVC envelopes were extracted from the Processed EMG and Processed MVC signals. The EMG envelope was normalized by the maximum value of the MVC envelope, giving the neural activation u(t) (Figure 2). Muscle activation a(t) was calculated using the model with the lowest data point optimization time [23], as suggested by Manal et Buchanan [37], given in Equation (1):

(1) $$a\left(t\right)=\frac{e^{\mathrm{Au}\left(t\right)}-1}{e^{A}-1}$$

where A = −20 was taken to achieve the range of muscle activation function [0,1].

An example of the specific measurement of the Processed EMG signal and its envelope data for E1 and E2 belonging to participant 9 is presented in Appendix A.

### 2.4. Algorithm for Elbow Joint Stiffness Estimation

**Figure 3:**
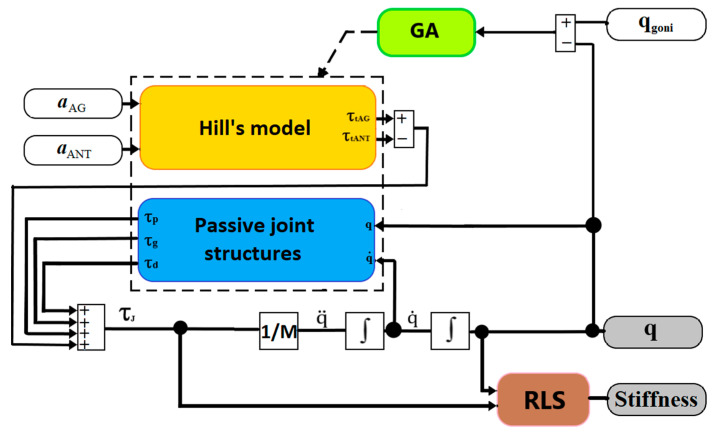
A block diagram of the joint stiffness estimation.

Figure 3 presents a block diagram for the overall process of elbow joint stiffness estimation based on Hill’s muscle model, the passive joint structure model, GA, and the Recursive Least-Squares (RLS) algorithm. The muscle activation function of the biceps brachii long head muscle ($a_{\mathrm{AG}}$(t)) and triceps brachii long head ($a_{\mathrm{ANT}}$(t)) are the inputs to Hill’s muscle model. The passive joint structure estimates passive torque (τ_p_), damping torque (τ_d_), and gravity torque (τ_g_) based on the estimated joint angle (q) and velocity ($\dot{q}$). Total joint torque (τ_J_) is defined as the sum of torques coming from the antagonistic muscle pair (τ_tAG,_ τ_tANT_) and passive joint structures (τ_p,_ τ_d,_ τ_g_). GA adjusts the parameters of Hill’s muscle model for the muscle pair and passive joint structures by taking into account the difference between the measured joint angle (q_goni_, using a goniometer) and the estimated joint angle (q). The RLS algorithm was used to estimate the joint stiffness (*Stiffness*) based on the estimated joint torque τ_J_ and the estimated joint angle q. The following sections will provide more details on all assessment modules.

#### 2.4.1. Hill’s Muscle Model

**Figure 4:**
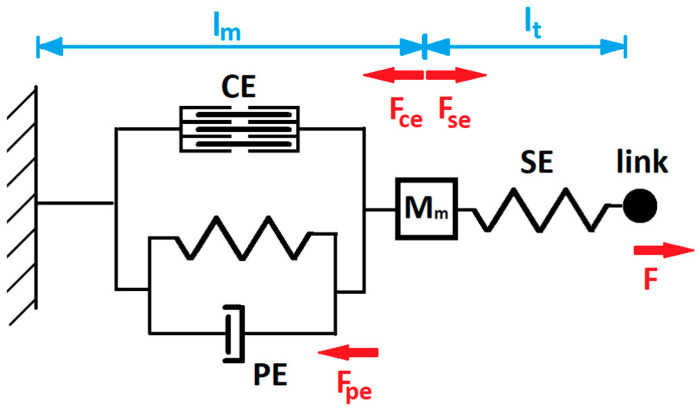
Hill’s muscle model.

A typical and widely used representation of the muscle is Hill’s muscle model [38] (Figure 4). The model contains: (1) a contractile element (CE) (a force generator imposed by neural activation u(t)), (2) a parallel element (PE) that has viscoelastic constants that oppose the movement, and (3) a series element (SE) that contributes to tendon elasticity. The total muscle mass is represented as the moment of inertia of the forearm–hand segment M. Neural activation u(t) in the muscle contractile element will shorten the fiber length of the muscle (l_m_), while the coupling force F will increase the length of the muscle.

The force generated by CE (F_CE_) is given in Equations (2)–(4):

(2) $$F_{\mathrm{CE}}=F_{\max}a\left(t\right)f\left(l_{m}\right)g\left(v_{m}\right)$$

(3) $$g\left(v_{m}\right)=\frac{1-v_{m}/v_{\max}}{1+\frac{1}{a_{f}}v_{m}/v_{\max}}$$

(4) $$f\left(l_{m}\right)=\exp\left(-{\left(\frac{\left(\frac{l_{m}}{l_{\mathrm{mopt}}}\right)-1}{w}\right)}^{2}\right)$$

where $F_{\max}$ is the maximum isometric force (the maximum force in the static state at MVC), a(t) is the muscle activation function, $v_{m}$ is the actual muscle velocity, $v_{\max}$ is the maximum unloaded shortening speed, $a_{f}$ is the Hill’s model parameter, $l_{m}$ is the actual muscle fiber length, $l_{\mathrm{mopt}}$ is the optimal muscle fiber length, and w is the width of the force–length relationship.

The total force $F_{\mathrm{PE}}$ of the PE element is the sum of the forces delivered by the stiffness (F_p_) and damping (F_d_) force (Equation (5)).

(5) $$F_{\mathrm{PE}}=F_{P}+F_{d}$$

The muscle can act as a spring and has a nonlinear characteristic. The spring nature of the muscle is modeled as stiffness with its force F_p_, given in Equations (6) and (7):

(6) $$F_{P}\left(l_{m}\right)=\{\begin{array}{cc} 0, & l_{m}<l_{\mathrm{ms}}\\ \frac{k_{\mathrm{ml}}}{k_{\mathrm{me}}}\left(\exp\left(k_{\mathrm{me}}\left(l_{m}-l_{\mathrm{ms}}\right)\right)-1\right), & l_{\mathrm{ms}}\leq l_{m}\leq l_{\mathrm{mc},}\\ F_{\mathrm{mc}}+k_{m}\left(l_{m}-l_{\mathrm{mc}}\right), & l_{m}>l_{\mathrm{mc}} \end{array}\;$$

(7) $$F_{\mathrm{mc}}=\frac{k_{\mathrm{ml}}}{k_{\mathrm{me}}}\left(\exp\left(k_{\mathrm{me}}\left(l_{\mathrm{mc}}-l_{\mathrm{ms}}\right)\right)-1\right)\;$$

where $l_{m}$ is the actual muscle fiber length, $l_{\mathrm{ms}}$ is the passive muscle slack length, $l_{\mathrm{mc}}$ is the length at which the spring becomes linear and is equal to the optimal muscle length $l_{\mathrm{mopt}}$, $k_{m}$ and $k_{\mathrm{ml}}$ are parallel spring constants, and $k_{\mathrm{me}}$ is an exponential shape parameter. The PE also contains a damping element for which the damping force F_d_ is given in Equation (8):

(8) $$F_{d}\left(v_{m}\right)=B_{m}v_{m}$$

where $B_{m}$ is the parallel damping constant and $v_{m}$ is the change in muscle speed over time.

The tendon connects muscle and bone and is modeled as SE. The force in the muscle tendon F_SE_ is given in Equations (9) and (10):

(9) $$F_{\mathrm{SE}}\left(l_{t}\right)=\{\begin{array}{cc} 0, & l_{t}<l_{\mathrm{ts}},\\ \frac{k_{\mathrm{tl}}}{k_{\mathrm{te}}}\left(\exp\left(k_{\mathrm{te}}\left(l_{t}-l_{\mathrm{ts}}\right)\right)-1\right), & l_{\mathrm{ts}}\leq l_{t}\leq l_{\mathrm{tc}}\\ F_{\mathrm{tc}}+k_{t}\left(l_{t}-l_{\mathrm{tc}}\right), & l_{t}<l_{\mathrm{tc}} \end{array}$$

(10) $$F_{\mathrm{tc}}=\frac{k_{\mathrm{tl}}}{k_{\mathrm{te}}}\left(\exp\left(k_{\mathrm{te}}\left(l_{\mathrm{tc}}-l_{\mathrm{ts}}\right)\right)-1\right)$$

where $k_{t}$ and $k_{\mathrm{tl}}\;$ represent tendon spring constants, $k_{\mathrm{te}}$ is the exponential shape parameter,${\;l}_{t}$ is the tendon length,${\;l}_{\mathrm{ts}}$ is the tendon slack length, and $l_{\mathrm{tc}}$ is the length at which the tendon becomes linear.

Having in mind muscle representation given in Equations (2)–(10), the actual muscle fiber length $l_{m}$, muscle speed $v_{m}$, and tendon length $l_{t}$ are given in Equations (11)–(13):

(11) $$v_{m}=\frac{1}{M_{m}}\left(F_{\mathrm{SE}}-F_{\mathrm{CE}}-F_{\mathrm{PE}}\right)$$

(12) $$l_{m}=\int_{0}^{t}v_{m}\mathrm{dt}$$

(13) $$l_{t}={\;l}_{\mathrm{ms}}+l_{\mathrm{ts}}+r_{p}q_{0}-l_{m}$$

where $M_{m}$ is the muscle mass, $r_{p}$ is the radius of the elbow joint calculated as a half value of the BSP parameter P10 from Table 1, and $q_{0}$ is the elbow joint angle during the full extension ($q_{0}=0\;\deg$).

The joint torque $\tau_{t}$ generated from the muscle and transmitted through the tendon $F_{\mathrm{SE}}$ via ${\;r}_{p}$ is given in Equation (14).

(14) $$\tau_{t}=F_{\mathrm{SE}}r_{p}$$

#### 2.4.2. Passive Joint Structures

Passive joint structure implies stability in the musculoskeletal model [39,40]. The passive joint torque $\tau_{p}$, the damping torque $\tau_{d}$, and the torque originating from gravity $\tau_{G}$ are given in t Equations (15)–(17):

(15) $$\tau_{p}\left(q\right)=k_{1}\exp\left(-k_{2}\left(q-q_{2}\right)\right)-k_{4}\exp\left(-k_{5}\left(q_{1}-q\right)\right)$$

(16) $$\tau_{d}\left(\dot{q}\right)=-B_{p}\;\dot{q}$$

(17) $$\tau_{G}={\mathrm{mgr}}_{\mathrm{cm}}\sin\left(\frac{\pi }{2}-q\right)$$

where m is the mass of the forearm–hand segment, $B_{p}$ is the joint damping constant, g is the gravitational constant, $r_{\mathrm{cm}}$ is the distance between the joint axis and the center of mass of the forearm–hand, $q_{1}$ and $q_{2}$ represent joint angles where the torque begins to increase,$\;k_{1}$ and $k_{4}$ are joint torque constants, and $\;k_{2}$ are $k_{5}$ are dimensionless parameters. The parameters $k_{1},{\;k}_{2}$, $k_{4}$, and $k_{5}\;$ define the shape of the torque joint curve given in Equation (15), which fits the torque joint curve experimentally defined and represented in [40].

The total joint torque $\tau_{J}$ is the sum of the torques originating from the passive joint structure and the torque induced by the antagonist’s muscle $\tau_{\mathrm{tAN}T}\;$ subtracted by the torque produced by the agonist’s muscle ${\;\tau }_{\mathrm{tAG}}$ (Equation (18)).

(18) $$\tau_{J}=\tau_{p}+\tau_{d}+\tau_{G}+\tau_{\mathrm{tAN}T}-\tau_{\mathrm{tAG}}$$

According to Newton’s Second Law for rotation, Equation (16), the joint angular velocity $\dot{q}$ and the joint angle q can be obtained through integration and double integration of the angular acceleration $\ddot{q}$, satisfying the initial static condition of the joints ${\dot{q}}_{0}=0$ and ${\;q}_{0}=0$ (Equations (19)–(21)):

(19) $$\tau_{J}=M\ddot{q}$$

(20) $$\;\dot{q}=\int_{0}^{t}\ddot{q}\mathrm{dt}+{\;\dot{q}}_{0}$$

(21) $$q=\int_{0}^{t}\;\dot{q}\mathrm{dt}+q_{0}$$

where M is the moment of inertia of the forearm–hand segment.

#### 2.4.3. GA for Adjustment of Parameters for Hill’s Muscle Model and Passive Joint Structure Model

GA is defined as a multi-objective optimization approach that aims to calculate the optimal combination of Hill’s and the passive joint structure model parameters (defined in Section 2.4.1 and Section 2.4.2) while considering the difference between the measured joint angle (q_goni_) and the estimated joint angle (q). Thus, one combination of coefficients in GA notation is one individual. Each individual consists of chromosomes and represents a unique solution within the solution space. For GA, in this paper, each chromosome represents one parameter. Accordingly, the initial values of these parameters and their ranges are defined and encoded into GA using the actual value of the chromosome representation. The population of the designed GA is represented by a set of 20 individuals. By testing GA with different numbers of individuals in one population, it was concluded that 20 individuals are a good compromise between the complexity of GA, its execution time, and optimization quality. To cover the complete solution space, a uniform distribution of individuals was used to generate the initial population. The fitness function defined by Equation (22) was evaluated for each individual per generation:

(22) $$F_{\mathrm{fitness}}=\int_{0}^{t_{\mathrm{end}}}\left(q_{\mathrm{goni}}-q\right)\mathrm{dt}\approx \frac{t_{\mathrm{end}}}{2N}\sum_{n=1}^{N}\left(q_{\mathrm{goni}}\left(t_{n}\right)-q\left(t_{n}\right)\right)$$

where q_goni_ is the joint angle measured using a goniometer, q is the estimated joint angle according to Equation (21), $t_{\mathrm{end}}$ is the motion duration, and N is the number of samples during the motion.

The value of the fitness function was used to determine the probability that each individual would be selected for the next generation during the selection process. Roulette-wheel selection was used, which selects the solutions with the lowest values of fitness function but also gives a chance to some weaker solutions to survive the selection process. A weaker solution may include some components that may prove useful after the crossover process. The roulette-wheel selection process was chosen to prevent convergence to a local minimum and to try to find a globally optimal solution. The crossover rate was chosen to be equal to 0.7, which means that 70% of new (child) individuals were created using the crossover operator. Other children were defined using the mutation operator or were elite individuals. Two individuals with the lowest values of the fitness function in the current generation (elite individuals) were selected and directly included in the next generation to accelerate the convergence of the GA to the optimal solution. To create new combinations of parameters (individuals) that differed from the existing ones, the mutation operator was used. Mutation provides genetic diversity, allows the GA to search a wider space, and can take individuals out of a local minimum and move them towards a global one. The mutation operator added a Gaussian distributed random value to a chromosome gene. If it fell outside the lower and upper limits for the gene, the new value of the gene was truncated. The imposed condition for stopping the GA was heuristically obtained since the change in the best value of the fitness function F was not greater than $10^{-6}$ for the previous 20 generations.

A PC platform with an Intel(R) Core(TM) i7-9700F CPU at 3.00 GHz and 64.0 GB RAM was used for the optimization procedure. The software used for the Hill’s and passive joint structure models was the Matlab2022 (license: Matlab Campus-Wide 2022) package Simulink. The Hill’s and passive joint structure model parameters were estimated using genetic algorithm functions *ga* within the Matlab Global Optimization toolbox. The *ga* function contained: an objective (fitness) function that is given in Equation (22); the number of variables set to 24 (the number of Hill’s and passive joint structure model parameters); ‘*PopulationSize*’ set to 20 individuals; ‘*SelectionFcn*’ set to ‘*selectionroulette*’; ‘*EliteCount*’ set to 2; and ‘*CrossoverFraction*’ set to 0.7. The values of the Hill’s and passive joint structure model parameters given in papers [19,20,21,22,23,24,25,26,27,28] were used to define the upper and lower bounds of the parameters. Half the value of the given parameters shown in the papers [19,20,21,22,23,24,25,26,27,28] was taken for the lower bound, while twice the value of the given parameters was taken for the upper bound. The CPU operating time was ~30 h for each participant and for each task.

#### 2.4.4. Verification of the Training GA Outputs

The measured joint angle (q_goni_) in the Training phase (described in Section 2.2) was used for verification of the GA estimation parameters from the Hill’s and passive joint structure models. The overall accuracy of the estimated parameters obtained by GA was obtained using the estimated joint angle (q) from the passive joint structure model for the values of the estimated parameters in the Training phase. The measured joint angle (q_goni_) from the Training phase and the estimated joint angle (q) were compared using Dynamic Time Wrapping (DTW) [41]. DTW gives the sum of the Euclidian distance between corresponding points of estimated joint angle (q) and the measured joint angle (q_goni_). The Training GA output verification is represented by the obtained DTW value divided by the number of samples.

#### 2.4.5. Recursive Least Square Algorithm

Analogously to the approach presented in [42,43], the RLS algorithm was applied to the estimated joint torque $\tau_{J}$ and joint angle q to estimate the elbow joint stiffness. The total joint torque $\tau_{J}$ was expressed as in Equation (23):

(23) $$\tau_{J}=\Phi \Pi$$

where $\Phi =\left(1,q,q^{2},\ldots ,q^{n}\right)$ is the n order torque regressor, which is the function of the joint angle, and $\Pi ={\left(\pi_{1},\pi_{2},\ldots \pi_{n}\right)}^{T}$ is a vector of the state parameters to be estimated. The order n=3 was chosen to capture the main features of the RLS mathematical model while the estimation was simultaneously denoised. The estimation of the state parameter Π is proposed in [44] and given by Equations (24)–(28):

(24) $$\epsilon \left[k\right]=\tau_{j}\left[k\right]-\Phi \left[k\right]\Pi \left[k\right]$$

(25) $$\rho \left[k\right]=\Phi^{T}\left[k\right]P\left[k-1\right]\Phi \left[k\right]$$

(26) $$G\left[k\right]={\left(1+\rho \left[k\right]\right)}^{-1}P\left[k-1\right]\Phi \left[k\right]$$

(27) Π[k]=Π[k−1]+G[k]ϵ[k]

(28) $$P\left[k\right]=P\left[k-1\right]-G\left[k\right]\Phi^{T}\left[k\right]P\left[k-1\right]$$

where ϵ[k] is the error of the measured ($\tau_{j}\left[k\right]$) and estimated torque values (Φ[k]Π[k]) in the kth time sample, P[k] is the covariance matrix, and G[k] is the gain of the RLS algorithm. The initial values of Π and P are given as $\Pi \left[0\right]={\left[0\;0\;0\right]}^{T}$ and $P\left[0\right]=0.01I_{3x3}$, where I is an identity matrix. The elbow joint stiffness (*Stiffness*) in each time sample k was calculated using Equation (29).

(29) $$Stiffness\;\left[k\right]=-\Phi {\left[k\right]}^{T}\Pi \left[k\right]$$

#### 2.4.6. Stiffness Functional Scale

The data acquired in E1 and E2 were used for designing novel functional scales. First, the parameters of the Hill’s and passive joint structure models estimated in the Training phase were applied to the acquired data in E1 and E2. Second, Dynamic Time Wrapping (DTW) [41] was performed on a measured joint angle (q_goni_) and the estimated joint angle (q) to quantify the estimation error. Finally, fitting curves (so-called functional scales) for different task frequencies (*Stiffness* (tempo) in E1) and different weights (*Stiffness* (weight) in E2) were defined.

## 3. Results

### 3.1. Identification of Hill’s and Passive Joint Structure Model Parameters

Table 2 presents the range (maximal and minimal values) of the parameter values of the Hill’s and passive joint structure models for elbow joints obtained using the GA algorithm. The muscle activation function of the biceps brachii long head muscle (a_AG_(t)) triceps brachii long head (a_ANT_(t)) and the measured elbow joint angle (q_goni_) from the Training phase were used as inputs in the Hill’s and passive joint structure models. The range values (minimum and maximum values) of the Hill’s and passive joint structure models’ parameters for the elbow joint for each parameter were calculated as the boundary values (minimum and maximum) of all values estimated for all participants for each parameter.

### 3.2. Verification of Hill’s Model and Passive Joint Structure Parameter Estimation

Verification of the accuracy of the Hill’s model and passive joint structure parameter estimation was performed using the DTW algorithm (defined in Section 2.4.4) and is presented in Table 3. The values in Table 3 represent the average deviation (in degrees) of the measured (q_goni_) and estimated (q) angles in the elbow joint (DTW divided by the number of samples) for 10 participants.

**Figure 5:**
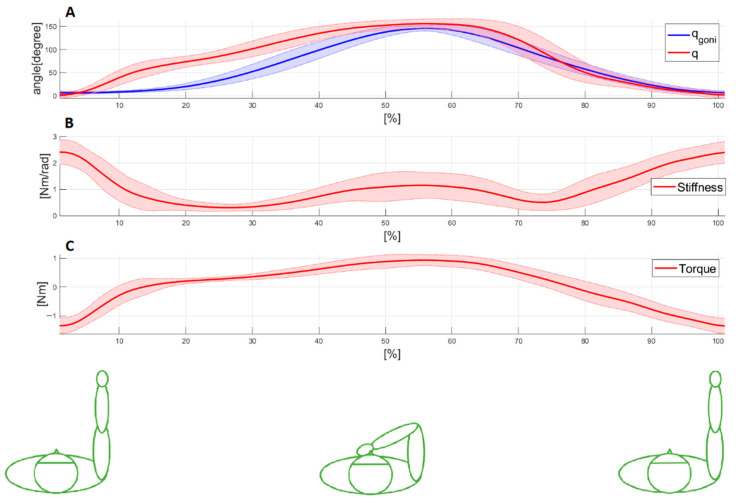
An example of WMFT8m movement pattern: mean value (bold line) and standard deviation (shaded area) of (**A**) measured and estimated elbow angle; (**B**) elbow joint stiffness; (**C**) total joint torque. The presented signals belong to participant 9 for task E1 and have a tempo of 30 bpm.

Figure 5 shows an example of the typical pattern for WMFT8_m_ movement. The mean value (bold line) and standard deviation (shaded area) of these signals were obtained by overlapping all WMFT8_m_ movements and their normalization with the time: (A) measured (q_goni_) and estimated (q) elbow joint angles; (B) estimated stiffness value (*Stiffness*); and (C) estimated total joint torque value ($\tau_{J}$).

The comparative analysis of stiffness accuracy in relation to the change in model parameter values is presented in Table 4. The values of the model parameters were modified for ±10%, ±20%, and ±30% regarding the parameter values estimated by GA. The accuracy of the stiffness was calculated using the DTW algorithm, comparing the estimated stiffness values using GA and stiffness calculated from modified parameters. The results show that stiffness sensitivity increases with the increase or decrease in each of the parameters. The stiffness is more sensitive to changes in the k_1_, k_2_, k_4_, q_2_, l_tc_, l_ts_, F_max_, l_mopt_, l_ms_, and v_max_ parameters (DWT ≥ 0.1) (see Table 4), while for the others, the change in stiffness is negligible. It can be observed that in the DWT value for l_tc_, l_ts_ is high, because the algorithm for stiffness diverged after parameter modification.

### 3.3. Elbow Joint Stiffness Estimation and Functional Scales

**Figure 6:**
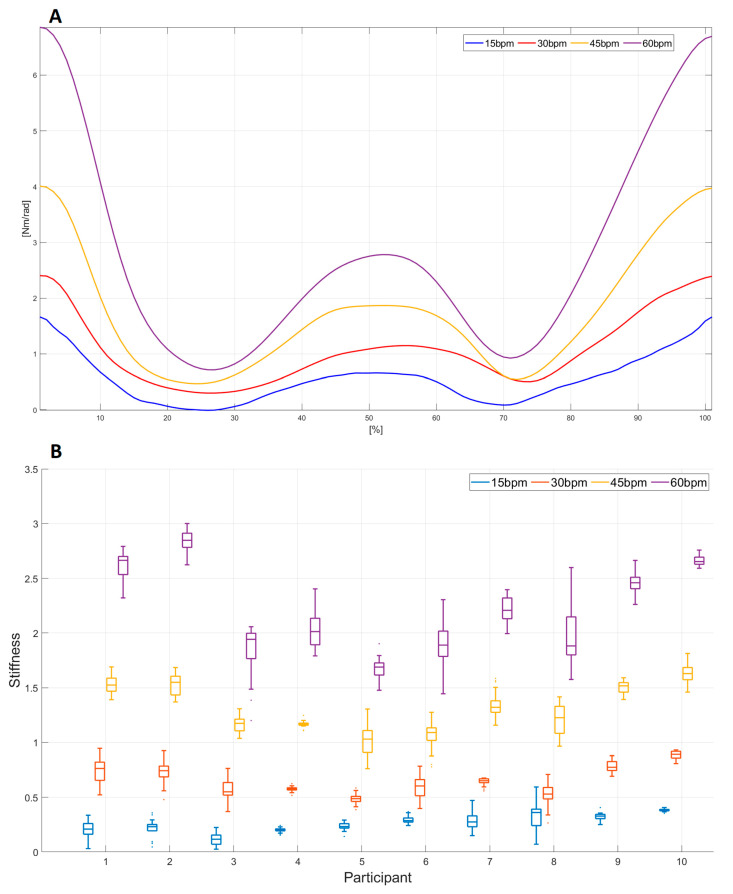
Comparative analysis of (**A**) elbow joint stiffness mean value for participant 9; (**B**) stiffness value for each tempo for all 10 participants in task E1.

The comparative analysis of the stiffness value through different tempos in task E1 is presented in Figure 6. The comparative analysis of the mean value of the stiffness signal for participant 9 for each tempo (15 bpm, 30 bpm, 45 bpm, and 60 bpm) is presented in Figure 6A. The stiffness mean values were calculated by overlapping all WMFT8_m_ movements and their normalization with the time. Using the boxplots, the stiffness behavior for each tempo for all 10 participants is shown in Figure 6B. Each boxplot represents the median and the interquartile range of the area under the stiffness signal (Figure 5B) for all repetitions of each participant.

**Figure 7:**
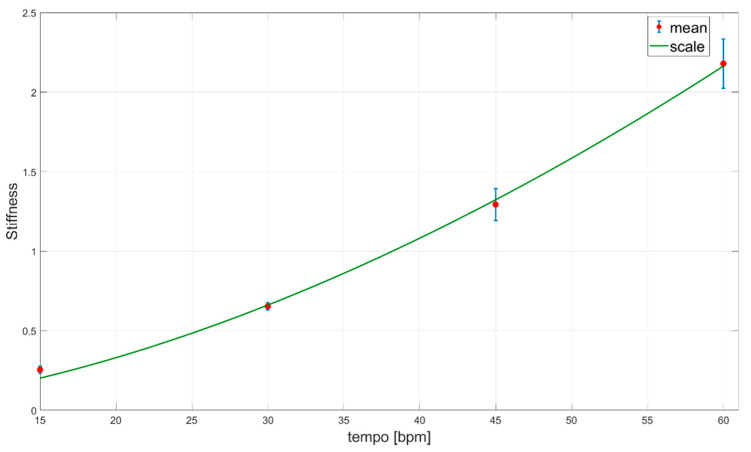
Stiffness fitting curve (green curve) for different task tempos (E1). Mean value for all participants is represented by red dots and the corresponding standard deviation is represented by blue lines.

A stiffness fitting curve (a so-called functional scale) for different task tempos Stiffness(tempo) is given in Equation (30) and shown in Figure 7.

(30) $$Stiffness\left(\mathrm{tempo}\right)=0.001975\cdot \;{\mathrm{tempo}}^{1.709}$$

**Figure 8:**
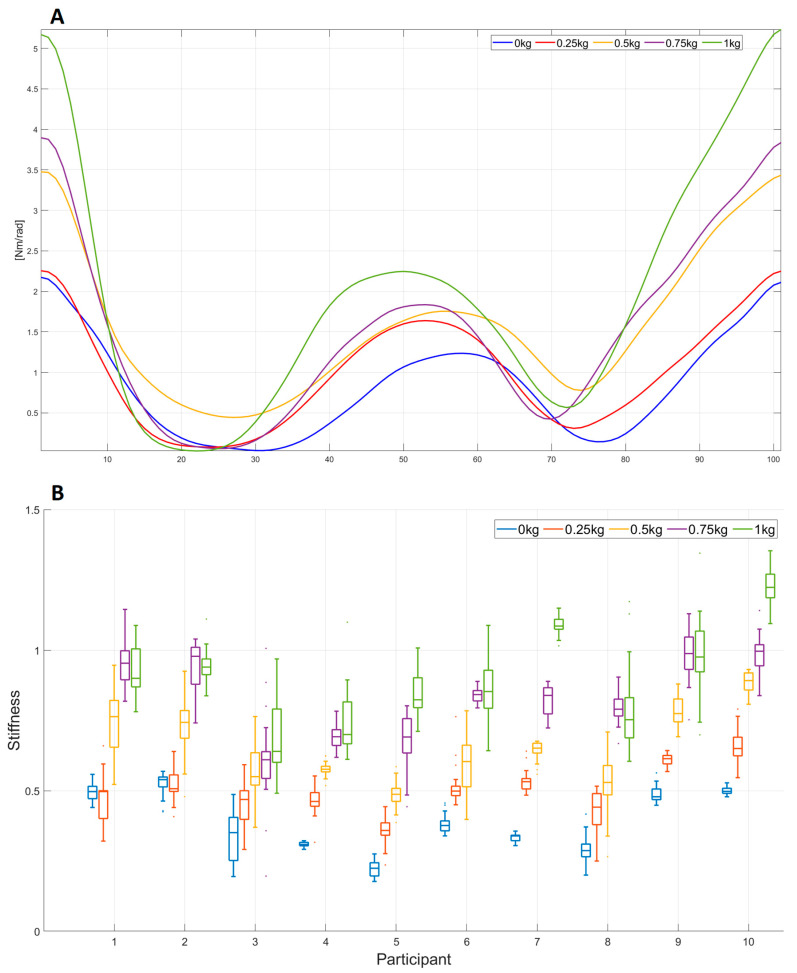
Comparative analysis of (**A**) elbow joint stiffness mean value for participant 9; (**B**) stiffness value for each tempo for all 10 participants in task E2.

The comparative analysis of the stiffness value through different loads in task E2 is presented in Figure 8. The comparative analysis of the mean value of the stiffness signal for participant 9 for each weight (0, 0.25, 0.5, 0.75, and 1 kg) is presented in Figure 8A. The stiffness mean values were obtained by overlapping all WMFT8_m_ movements and their normalization with the time. Using the boxplots, the stiffness behavior for each load for all 10 participants is shown Figure 8B. Each boxplot represents the median and the interquartile range of the area under the stiffness signal (Figure 5B) for all repetitions of each participant.

**Figure 9:**
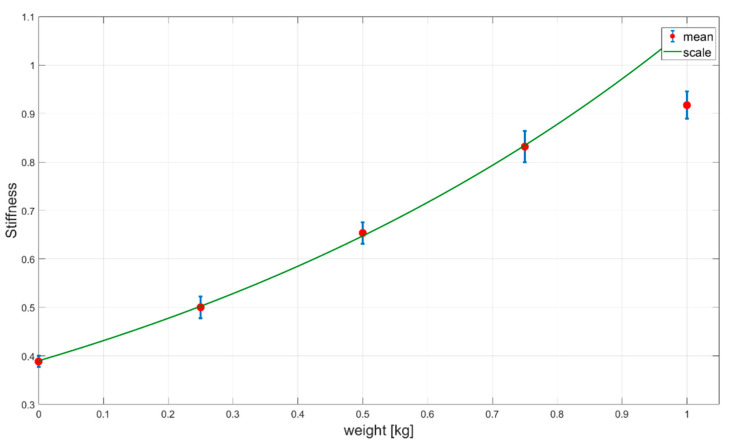
Stiffness fitting curve (green curve) for different “pulling” weights task (E2). Mean value for all participants is presented by red dots and corresponding standard deviation is presented by blue lines.

Stiffness fitting curves (so-called functional scales) for different “pulling” weights Stiffness(weight) are given in Equation (31) and shown in Figure 9.

(31) Stiffness(weight)=0.39· exp(1.014· weight).

## 4. Discussion

This paper presents a novel methodology for elbow joint stiffness estimation in people without sensorimotor disorders during different movement scenarios regarding movement tempo and the upper limb load. The overall research goal of the paper is to define a *Stiffness* functional scale (general *Stiffness* curve pattern) that is useful for the further neurorehabilitation follow-up of patients.

Considering that the musculoskeletal system of the upper limb is a nonlinear model, Hill’s muscle model and the passive joint structure model are convenient for use in such research [18,19]. One of the challenges when using Hill’s muscle model and passive joint structure is the estimation of the model parameters, which are the physical parameters of the muscles and tendons themselves, as well as the parameters that describe the characteristics of their behavior during movement. To the best of our knowledge, no report includes a complete list of parameter values for the antagonistic pair (the triceps brachii long head and biceps brachii long head muscle) and passive joint structure. Parameter values that describe the lengths of the above-mentioned muscles (l_mopt_, l_ts_, l_ms_) are presented in [20,21,22,23,24,25,26,27,28]; additionally, F_max_ values are given in [20,21,23,24,27,28], $M_{m}$ values are given in [22,27], $a_{f}$ values are presented in [22], and $v_{\max}\;$ values are given in [22,28]. In this paper, we proposed an experimental task corresponding to the clinical Wolf test, which aims to assess human/patient capabilities. The task comprises the repetition of a movement with different velocities and loads. To the best of our knowledge, there has been no similar scenario performed in the literature, and this fact makes comparison with previous research difficult. Additionally, it is worth remarking that exact equivalence among parameters is impossible due to the high variability in the human body. However, good matching is observed for the parameters l_mopt_, l_ts_, l_ms_, F_max_, $M_{m}$, $a_{f}$, and $v_{\max}$, as presented in Table 5.

Cavallaro et al. [20] and Zhao et al. [45] used GA in their work to estimate a subset of Hill’s model parameters. Other parameters of the Hill model, as well as all parameters of the passive joint structure for the elbow joint, are not described in the literature, so one of the major contributions of this paper is the presentation of the complete estimation of all parameters of the Hill’s muscle model and the passive joint structure model using GA. The range values (minimum and maximum values) of all the parameters estimated in the Training phase of ten participants are presented in Table 2. Considering that during the experiment, the tempo of the WMFT8m movement was variable (see Section 2.2), different dynamics of the elbow joint movement were taken into account during parameter estimation. The quality of parameter estimation using GA was tested using the DTW algorithm by comparing acquired and estimated values in the elbow joint. The values of the DTW analysis show that the maximum deviation is 9.16 deg, which corresponds to 6.1% of the full elbow angle range values during one WMFT8m movement (see Table 3). The parameters estimated from the Training phase were applied in experiments E1 and E2 for all participants to estimate stiffness in the elbow joint. A maximum deviation of 12.16 deg, which corresponds to 8.1% of the full elbow angle range values during one WMFT8m movement, was obtained for E1 and E2 (see Table 3). Additionally, a visual representation of the pattern matching of the estimated and acquired elbow joint angle is presented in Figure 5A. Stiffness in the elbow joint for participant 9 in task E1 and task E2 is presented in Figure 6A and Figure 8A, respectively. The maximum values of the estimated stiffness for task E1 are in the range [1.75 Nm/rad–7 Nm/rad], while those for task E2 are in the range [2.2 Nm/rad–5.25 Nm/rad]. Obtained maximal values of joint stiffness are aligned with the values of elbow joint stiffness presented in papers [15] (from 3 to 20 Nm/rad), [46] (from 2.5 to 9 Nm/rad), [47] (from 9 to 14 Nm/rad), and [48] (~10 Nm/rad).

Due to the variability in biological mechanisms and experimental conditions, we qualitatively compared the stiffness evolution in our work with results from the relevant literature in cases where the human arm is moving with different velocities and where it is acting against different forces. Humans perform interaction tasks with ease and precision by modulating their mechanical stiffness. It is intuitively expected and proven in the literature that the co-contraction of antagonistic muscle groups (which is highly related to joint mechanical impedance) depends on velocity [49] or load [50]. However, for a number of challenges that include human motion analysis, rehabilitation, and human–robot interaction, comprehensive study and understanding of the mutual influence of velocity and load on human motion and stiffness planning are needed. Furthermore, considering the latest enabling technology—collaborative robots capable of mechanical impedance modulation—the impact that cobots can have on healthcare and industry highly depends on our capabilities to understand human behavior patterns in contact and non-contact tasks.

The stiffness estimation in tasks E1 and E2 is presented in Figure 6, Figure 7, Figure 8 and Figure 9. To the best of our knowledge, no report analyzes the stiffness in such a way in the existing literature. The obtained results for experiment E1 (Figure 6) show that the stiffness value increases with the increase in the movement tempo and that the same effect was observed in all participants. Therefore, it can be concluded that there is a universal relationship that connects the stiffness and the movement tempo, and this dependence can be represented by Equation (30). The corresponding curve is shown in Figure 7 and it represents the functional scale of the stiffness change regarding the movement tempo change in the population without sensorimotor disorders. The obtained functional scale can be used as a reference curve for assessing the recovery of patients with upper extremity weakness. Compared to the papers [46,49], which analyze the correlation between joint stiffness and joint velocity, we obtained quite similar results: the increase in speed affects the increase in stiffness. A similar stiffness-increasing trend with increasing weight in the E2 task is presented in Figure 8. It can be observed that in participants 5, 7, and 10, stiffness has an increasing trend for all increasing weights, including 1 kg. Upon comparing the stiffness–force relationship with the results in [8], it can be noticed that with increasing force, stiffness also increases, which complies with the conclusions of [8]. However, in most cases (participants 1, 2, 3, 4, 6, 8, and 9), stiffness has an increasing trend for all increasing weights except for 1 kg, where the stiffness value is close to the stiffness at 0.75 kg. A possible reason for this phenomenon is the saturation of stiffness in the elbow joint with an increase in weights greater than 0.75 kg. This saturation property of elbow joint stiffness can be explained by the compensatory ability of motor behavior [47]. Based on the obtained results for different weights, the functional scale of stiffness change regarding weight change can be defined and is given in Equation (31). The corresponding curve is given in Figure 9. Due to the previously mentioned saturation effect, the stiffness deviation at 1 kg is larger than for other weights. Considering that the 0.5 kg weight is conventionally used in task 8 of WMFT, the obtained functional scale can also be used as a reference for assessing the recovery of patients with upper extremity weakness [51].

## 5. Conclusions

This paper introduced a general assessment methodology for elbow joint stiffness estimation based on Hill’s muscle model and the passive joint structure model, and a genetic optimization procedure during the “Reach and retrieve” task that is conventionally used within neurorehabilitation testing. The model parameters were estimated using the short-term tempo ramp scenario, and then, verified in two independent scenarios, resulting in 6.1% and 8.1% errors for the training and test datasets, respectively. The estimated joint stiffness in the two long-term test scenarios (the first one with tempo variation and the second one with load variation) was used for creating elbow stiffness curves to represent the general pattern of motor behavior in the population without sensorimotor disorders. The proposed objectification criteria of motor behavior in the form of stiffness patterns could significantly contribute to the monitoring of motor recovery in patients with sensorimotor disorders. A comparison to the results obtained using the proposed model with existing clinical scales (for the selected task as well as for the overall functional assessment of the arm) would be of interest for future work, as would further automatization of the overall system using collaborative robot-based approaches. Understanding human stiffness patterns and stiffness monitoring is a prerequisite for the development of rehabilitation devices based on collaborative robots capable of imposing recommended stiffness patterns, on the one hand, and monitoring Cartesian stiffness in contact tasks, on the other hand. Future research will exploit the presented methodology in clinical studies for assessment of the rehabilitation process with a larger number of participants.

## Figures and Tables

**Table 1:** Selected body segment parameters (BSP).

No	Parameter	No	Parameter
1	Length, Hand	6	Circumference, Forearm
2	Length, Wrist to Knuckle	7	Circumference, Elbow
3	Length, Forearm	8	Width, Hand
4	Circumference, Fist	9	Width, Wrist
5	Circumference, Wrist	10	Width, Elbow

**Table 2:** Parameters of Hill’s model and passive joint structure model for the elbow joint.

Parameter	Mean Value ± Standard Deviation	Min Value	Max Value
k_1_ [Nm]	7.16 ± 1.88	4.54	9.05
k_2_ [${\mathrm{rad}}^{-1}$]	0.35 ± 0.05	0.25	0.43
k_4_ [Nm]	35.89 ± 13.24	15.00	60.5
k_5_ [${\mathrm{rad}}^{-1}$]	0.026 ± 0.006	0.02	0.04
q_1_ [rad]	4.20 ± 0.77	3.11	5.49
q_2_ [rad]	5.25 ± 1.29	3.22	6.95
B_p_ [Nms]	3.32 ± 0.81	1.55	4.05
F_max_ [N]	2289.76 ± 786.18	1401.59	3913.00
w [n.u.]	0.78 ± 0.14	0.64	0.96
l_mopt_ [m]	0.19 ± 0.05	0.10	0.27
a_f_ [n.u.]	0.39 ± 0.19	0.25	0.75
k_te_ [$m^{-1}$]	2611.95 ± 862.11	1511.02	4042.59
k_tl_ [N/m]	27,230.04 ± 8846.59	15,570.31	43,133.01
k_t_ [N/m]	538,599.51 ± 196,458.08	317,771.71	923,909.99
l_tc_ [m]	0.34 ± 0.093	0.23	0.47
l_ts_ [m]	0.33 ± 0.070	0.24	0.46
k_ml_ [N/m]	408.21 ± 137.80	244.96	710.60
k_me_ [$m^{-1}$]	75.84 ± 23.35	46.05	113.92
k_m_ [N/m]	4551.31 ± 1590.63	2500.06	7352.47
l_mc_ [m]	0.14 ± 0.03	0.10	0.19
l_ms_ [m]	0.19 ± 0.05	0.10	0.27
B_m_ [Ns/m]	0.19 ± 0.05	130.97	342.17
$M_{m}\;\left[\mathrm{kg}\right]$	241.77 ± 75.83	0.33	0.45
$v_{\max}\;\left[m/s\right]$	0.39 ± 0.04	0.54	1.46

**Table 3:** Verification of the Training GA outputs using DTW—average deviation (in degrees).

		1	2	3	4	5	6	7	8	9	10
Training phase		8.47	4.46	8.02	1.95	8.67	9.16	4.89	2.56	2.77	1.83
E1	15 bpm	2.78	5.21	8.43	4.37	5.42	5.48	6.67	1.63	3.55	5.80
	30 bpm	4.45	1.98	2.91	4.05	5.16	5.83	1.78	5.25	2.96	2.16
	45 bpm	4.80	3.11	6.05	2.06	6.32	6.51	2.43	2.23	2.95	2.31
	60 bpm	5.31	5.74	10.79	6.56	11.85	7.03	3.13	4.76	3.65	0.99
E2	0 kg	4.55	7.80	8.47	2.52	4.35	12.16	11.43	5.10	4.75	6.94
	0.25 kg	4.76	6.55	6.64	4.91	5.33	3.53	10.15	6.29	2.23	3.02
	0.5 kg	4.45	1.98	2.91	4.05	5.16	5.83	1.78	5.25	2.96	2.16
	0.75 kg	3.70	7.22	11.22	3.20	3.93	8.65	5.49	1.69	2.93	11.14
	1 kg	8.13	5.44	10.79	4.38	5.35	8.82	5.32	6.96	2.30	3.26

**Table 4:** Verification of the accuracy of the generated stiffness patterns depending on the change in parameter values using DTW—average deviation (in Nm/rad).

Parameter	Percentage Variation					
	−10%	+10%	−20%	20%	−30%	30%
k_1_	0.0622	0.0429	0.1784	0.1370	0.2854	0.2916
k_2_	0.1712	0.1341	0.3541	0.3405	0.4556	0.5504
k_4_	0.0331	0.0353	0.1078	0.0916	0.2543	0.1592
k_5_	0.0019	0.0019	0.0040	0.0038	0.0064	0.0059
q_1_	0.0024	0.0024	0.0052	0.0050	0.0084	0.0077
q_2_	0.1669	0.1934	0.3211	0.3747	0.4050	0.3827
B_p_	0.0041	0.0040	0.0100	0.0094	0.0173	0.0155
F_max_	0.0239	0.0243	0.0538	0.0614	0.1028	0.1066
w	0.0142	0.0104	0.0446	0.0235	0.0994	0.0367
l_mopt_	0.0444	0.0287	0.0938	0.0548	0.1887	0.0899
a_f_	0.0193	0.0159	0.0559	0.0377	0.0994	0.0609
k_te_	0.00035	0.00042	0.0005	0.00064	0.00057	0.00072
k_tl_	0.00028	0.00033	0.00037	0.00036	0.00047	0.00047
k_t_	0.00074	0.00071	0.0012	0.00087	0.0020	0.0011
l_tc_	5.0826	0.0038	7.1025	0.0038	5.8225	0.0038
l_ts_	0.0038	4.9648	0.0038	6.9452	0.0038	6.2682
k_ml_	0.00028	0.00031	0.00043	0.00044	0.0005	0.00051
k_me_	0.00032	0.00026	0.00046	0.00039	0.00056	0.00046
k_m_	0.0074	0.0072	0.0186	0.0168	0.0322	0.0277
l_mc_	0.0082	0.0133	0.0110	0.0215	0.0114	0.0298
l_ms_	0.0444	0.0287	0.0938	0.0548	0.1887	0.0899
B_m_	0.00079	0.00079	0.0013	0.0012	0.0018	0.0018
$M_{m}\;$	0.00005	0.00009	0.00007	0.00014	0.00009	0.00017
$v_{\max}\;$	0.0254	0.0215	0.0733	0.0517	0.1440	0.0846

**Table 5:** Comparative analysis of the Hill’s muscle model and passive joint structure model parameters.

Reference	l_mopt_ [m]	l_ts_ [m]	l_ms_ [m]	F_max_ [N]	M_m_ [kg]	a_f_ [n.u.]	v_max_ [m/s]
Cavallaro et al. [20]	0.15	0.19–0.23	n.a.	393–1000	n.a.	n.a.	n.a.
Garner et al. [21]	0.15	0.19–0.23	n.a.	462–629	n.a.	n.a.	n.a.
Winters et al. [22]	0.09–0.14	0.22	0.31–0.37	n.a.	0.06–0.13	0.35–0.45	0.49–0.76
Desplenter et al. [23]	0.11–0.17	n.a.	0.16–0.25	2875–2397	n.a.	n.a.	n.a.
Holzbaur et al. [24]	0.11–0.13	0.14–0.27	n.a.	624–799	n.a.	n.a.	n.a.
Murray et al. [25]	0.13	0.22–0.23	0.22–0.36	n.a.	n.a.	n.a.	n.a.
Yu et al. [26]	0.15	0.11–0.13	n.a.	n.a.	n.a.	n.a.	n.a.
Song et al. [27]	0.16–0.17	0.21–0.23	0.39–0.41	630–764	0.34–0.45	n.a.	n.a.
Bingshan et al. [28]	0.12–0.13	0.14–0.27	n.a.	624–799	n.a.	n.a.	0.96–1.04
This paper	0.1–0.27	0.24–0.46	0.1–0.27	1400–3900	0.33–0.45	0.25–0.75	0.54–1.46

**Figure A1:**
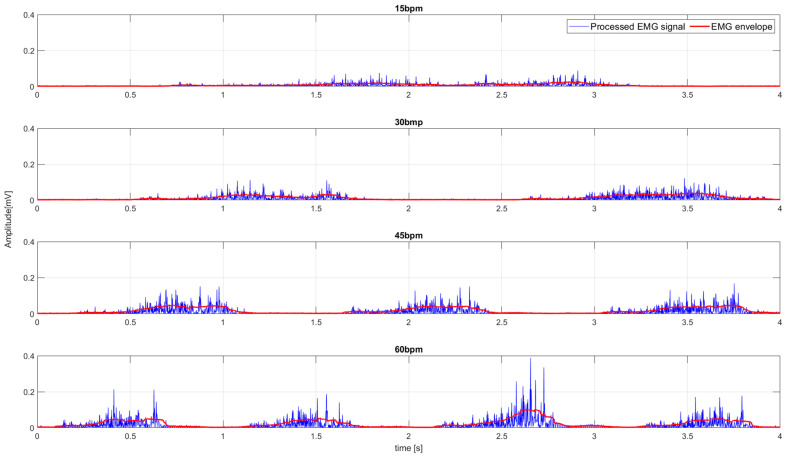
Muscle activation estimation: Processed EMG signal (blue line) and EMG envelope (red line) in task E1.

**Figure A2:**
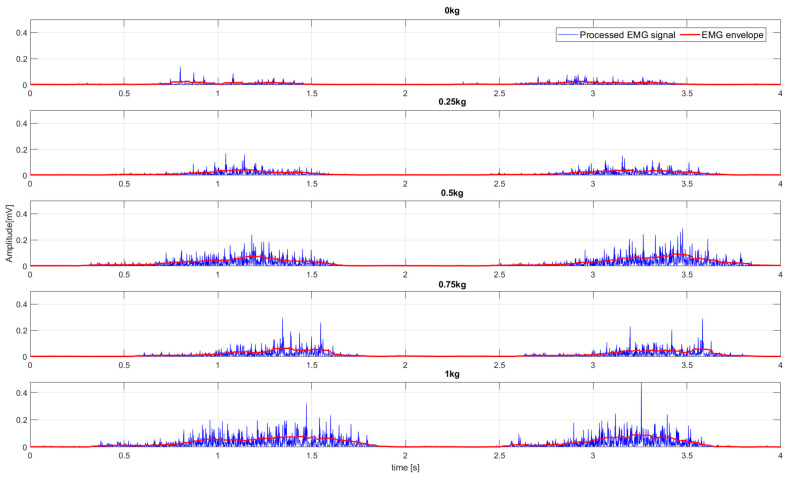
Muscle activation estimation: Processed EMG signal (blue line) and EMG envelope (red line) in task E2.

## Data Availability

All information is given on project official website http://fornextcobot.etf.bg.ac.rs/sr/naslovna/en/naslovna-english/.

## Appendix A

The specific measurement data (Processed EMG signal and its envelope) for tasks E1 and E2 for participant 9 are presented in Figure A1 and Figure A2, respectively. The results for task E1 are presented in Figure A1. The EMG signals and envelopes are represented for a time sequence of 4 s. The different tempo of the motion is evident in the EMG signals and envelopes. For a tempo of 15 bpm during 4 s, the motion is performed once, while for a tempo of 60 bpm during 4 s, the motion is performed four times. Additionally, increasing the tempo affects the values of the Processed EMG signal and its envelope (see Figure A1). The maximal envelope value for a tempo of 15 bpm is ~0.02 mV, for a tempo of 30 bpm is ~0.03 mV, for a tempo of 45 bpm is 0.05 mV, and for a tempo of 60 bpm is 0.1 mV.

In task E2, increasing the load affects the values of the Processed EMG signal and its envelope (see Figure A2). The EMG signals and envelopes are represented for the time sequence of 4 s. Because task E2′s tempo is 30 bpm, the motion is repeated four times for 4 s for each load. The results show that the EMG signals and envelope increase with increased load. The maximal envelope value for a load of 0 kg is ~0.025 mV, for a load of 0.25 kg is ~0.04 mV, for a load of 0.5 kg is ~0.06 mV, for a load of 0.75 kg is ~0.07 mV, and for a load of 1 kg is ~0.1 mV.
